# Intranasally administered S-MGB-364 displays antitubercular activity and modulates the host immune response to *Mycobacterium tuberculosis* infection

**DOI:** 10.1093/jac/dkac001

**Published:** 2022-01-25

**Authors:** Nathan S. Kieswetter, Mumin Ozturk, Lerato Hlaka, Julius Ebua Chia, Ryan J. O. Nichol, Jasmine M. Cross, Leah M. C. McGee, Izaak Tyson-Hirst, Rebecca Beveridge, Frank Brombacher, Katharine C. Carter, Colin J. Suckling, Fraser J. Scott, Reto Guler

**Affiliations:** 1 International Centre for Genetic Engineering and Biotechnology, Cape Town Component, Cape Town 7925, South Africa; 2 Department of Pathology, University of Cape Town, Institute of Infectious Diseases and Molecular Medicine (IDM), Division of Immunology and South African Medical Research Council (SAMRC) Immunology of Infectious Diseases, Faculty of Health Sciences, University of Cape Town, Cape Town 7925, South Africa; 3 Department of Pure and Applied Chemistry, University of Strathclyde, Glasgow G1 1XL, Scotland; 4 Wellcome Centre for Infectious Diseases Research in Africa (CIDRI-Africa), Institute of Infectious Disease and Molecular Medicine (IDM), Faculty of Health Sciences, University of Cape Town, Cape Town 7925, South Africa; 5 Strathclyde Institute of Pharmacy of Biomedical Sciences, University of Strathclyde, Glasgow G4 0NR, Scotland

## Abstract

**Background:**

Previously, we evaluated the intracellular mycobactericidal activity of the minor groove binder, S-MGB-364 against the clinical *Mycobacterium tuberculosis* (Mtb) strain HN878 in macrophages.

**Objectives:**

To assess the mycobactericidal activity of S-MGB-364 in Mtb-infected mice. Further, we investigated a plausible DNA binding mechanism of action of S-MGB-364.

**Methods:**

The anti-TB and host immune effects of intranasal S-MGB-364 or S-MGB-364 encapsulated in non-ionic surfactant vesicles (NIV) were assessed in Mtb-infected mice by cfu enumeration, ELISA, histology, and flow cytometry. DNA binding was examined using native mass spectrometry and UV-vis thermal melt determination. S-MGB interference with DNA-centric biological events was assessed using a representative panel of Mtb and human topoisomerase I, and gyrase assays.

**Results:**

S-MGB-364 bound strongly to DNA as a dimer, significantly increasing the stability of the DNA:S-MGB complex compared with DNA alone. Moreover, S-MGB-364 inhibited the relaxation of Mtb topoisomerase I but not the human form. In macrophages, S-MGB-364 or S-MGB-364-NIV did not cause DNA damage as shown by the low γ-H2AX expression. Importantly, in the lungs, the intranasal administration of S-MGB-364 or S-MGB-364-NIV formulation in Mtb-infected mice was non-toxic and resulted in a ∼1 log cfu reduction in mycobacterial burden, reduced the expression of proinflammatory cytokines/chemokines, altered immune cell recruitment, and importantly reduced recruitment of neutrophils.

**Conclusions:**

Together, these data provide proof of concept for S-MGBs as novel anti-TB therapeutics *in vivo*.

## Introduction

Minor groove binders (MGBs) have great potential as anti-infective agents.^1^ In particular, analogues of distamycin and netropsin synthesized at the University of Strathclyde, Strathclyde Minor Groove Binders (S-MGBs) have been shown to possess *in vivo* and *in vitro* activity against fungi,^2^ parasites,^3^ and Gram-positive bacteria.^4^ Their mechanism of action involves engagement at multiple DNA sites in the pathogen, and interference with normal protein synthesis at promoter sites in the case of Gram-positive bacteria.^5^ One S-MGB, MGB-BP-3, is ready for Phase 3 clinical trials for the treatment of *Clostridioides difficile* infection, having shown clinical potential.^6^ Although binding to AT-rich sites in dsDNA has been shown to be a key component of the mechanism of action,^4,5^ the key to selectivity, and therefore, utility as a drug, relies upon the physicochemical properties of MGBs.

We previously reported that S-MGBs have direct mycobactericidal activity against *Mycobacterium tuberculosis* (Mtb).^2,7^ Significantly, most of the active compounds contained S-MGBs with amidine tail groups. In particular, S-MGB-362 and S-MGB-364 had intracellular activity against Mtb in macrophages.^7^ This activity was enhanced by incorporating S-MGBs in non-ionic surfactant vesicles (NIV).^7^ Previous studies have shown that NIV can increase the efficacy of drugs by different routes, including inhalation, where NIV increased the activity of amphotericin B against *Aspergillus* in a murine model.^8^

In this study, we demonstrate that intranasally administered S-MGB-364 in mice has anti-Mtb activity and that S-MGB-364 did not induce genotoxicity. Mechanistically, we demonstrated that S-MGB-364 strongly binds to DNA as a dimer and inhibits the action of Mtb topoisomerase I relaxation.

## Materials and methods

### Mice

Male C57BL/6 mice were purchased from Jackson Laboratories (USA) and housed in a biosafety level 3 containment facility as previously described.^9^

### Ethics

All animal experiments were conducted in accordance with the Animal Research Ethics Committee of South African National Standard (SANS 10386:2008). The protocol (019/24) was approved by the Animal Ethics Committee, Faculty of Health Sciences, University of Cape Town, South Africa.

### UV-Vis DNA thermal melting experiments

Salmon genomic DNA (gDNA; D1626, Sigma–Aldrich) at 1 mg/mL in 1 mM phosphate buffer (pH 7.4) containing 0.27 mM KCl and 13.7 mM NaCl (P4417, Sigma–Aldrich) was annealed at 90°C for 10 min. S-MGBs at 10 mM in DMSO were diluted with the same phosphate buffer to yield a single sample with 10 μM S-MGB and 0.02 mg/mL gDNA in 1 mM phosphate buffer containing 0.27 mM KCl and 13.7 mM NaCl. Control samples containing only S-MGB or gDNA were prepared, respectively. Samples were melted at a rate of 0.5°C/min from 45°C to 90°C with spectra recorded at 260 nm on a UV-1900 UV-vis spectrophotometer fitted with a Peltier temperature controller (Shimadzhu) using LabSolutions (Tm Analysis) software. The melting temperatures (T_m_s) of the S-MGB:DNA complexes were determined by fitting a sigmoidal function using a Boltzmann distribution in OriginPro. Two independent experiments were carried out with values quoted with an error no worse than ±1°C.

### Native mass spectrometry

The purity of lyophilized DNA oligonucleotide sequence 5′-CGCATATATGCG-3′ (Alpha DNA, Canada) was confirmed by NMR. 100 μM DNA stock solutions were prepared with 150 mM ammonium acetate buffer solution and 2 mM KCl solution (Fisher Scientific, Loughborough, Leicestershire, UK). This solution was annealed at 90°C for 10 min and allowed to cool to room temperature. 10 mM S-MGB stocks in 100% DMSO were diluted to 1 mM S-MGB solution with 150 mM ammonium acetate to yield final concentrations of 9 μM DNA, 100 μM KCl, and 100 μM S-MGB, 1% DMSO. DNA solutions containing no S-MGB including 1% DMSO were used as controls.

Native mass spectrometry (nMS) experiments were carried out on a Synapt G2-Si instrument (Waters, Manchester, UK) with a nanoelectrospray ionization source (nESI). Mass calibration was performed by a separate infusion of NaI cluster ions. Solutions were ionized from a thin-walled borosilicate glass capillary (i.d. 0.78 mm, o.d. 1.0 mm, (Sutter Instrument Co., Novato, CA, USA) pulled in-house to nESI tip with a Flaming/Brown micropipette puller (Sutter Instrument Co., Novato, CA, USA). A negative potential in the range of 1.0–1.2 kV was applied to the solution via a thin platinum wire (diameter 0.125 mm, Goodfellow, Huntingdon, UK). The following instrument parameters were used for the DNA:S-MGB-364 complex: capillary voltage 1.2 kV, sample cone voltage 80 V, source offset 110 V, source temperature 40°C, trap collision energy 3.0 (V), trap gas 4 mL/min. For DNA:S-MGB-176 complex: capillary voltage 1.1 kV, sample cone voltage 90 V, source offset 110 V, source temperature 40°C, trap collision energy 3.0 (V), trap gas 4 mL/min was used. For DNA with no S-MGB present, a capillary voltage of 1.0 kV was applied to the sample. Sample cone voltage 80 V, source offset 95 V, source temperature 40°C, trap collision energy 3.0 (V) and trap gas 4.0 mL/min was used. Data were processed using Masslynx V4.2 and OriginPro 2021, and figures were produced using Chemdraw.

### Topoisomerase and gyrase inhibition

The activity of each enzyme was determined, and 1 U enzyme was required to fully supercoil or relax the substrate. Compounds ranging from 0.01 μM to 100 μM were added to the reaction before the addition of the enzyme. The final DMSO concentration in the assays was 1% (v/v).

#### M. tuberculosis and human topoisomerase I relaxation assay

1 U of Mtb and human topoisomerase I (topo I) was incubated with 0.5 μg supercoiled plasmid DNA (pBR322) in a 30 μL reaction at 37°C for 30 min under the following conditions: 40 mM Tris-HCl (pH7.6), 20 mM NaCl, 1 mM EDTA, 5 mM MgCl_2_ and 0.05 mg/mL BSA for Mtb topo I and 20 mM Tris HCl (pH 7.5), 200 mM NaCl, 0.25 mM EDTA and 5% glycerol plus 5% DMSO for human topo I. Each reaction was stopped using 30 μL chloroform/iso-amyl alcohol (24:1) and 30 μL Stop Dye before being loaded on a 1.0% TAE gel run at 90 V for 2 h.

#### M. tuberculosis gyrase supercoiling assay

1 U of DNA gyrase was incubated with 0.5 μg of supercoiled pBR322 DNA in a 30 μL reaction at 37°C for 30 min under the following conditions: 50 mM HEPES, KOH (pH 7.9), 6 mM magnesium acetate, 4 mM DTT, 1 mM ATP, 100 mM potassium glutamate, 2 mM spermidine and 0.05 mg/mL BSA. Each reaction was stopped using 30 μL chloroform/iso-amyl alcohol (24:1) and 20 μL Stop Dye (40% sucrose, 100 mM Tris-HCl (pH 7.5), 10 mM EDTA, 0.5 μg/mL bromophenol blue), before being loaded on a 1.0% TAE (Tris-acetate 0.04 mM, EDTA 0.002 mM) gel run at 80 V for 3 h.

### Preparation of S-MGBs and non-ionic surfactant vesicles

S-MGB-364 was reconstituted in DMSO and then diluted in 1×PBS or DMEM to yield a final concentration of 10 mg/kg (S-MGB-364) per mouse or 10 μM in macrophages. Freeze-dried NIVs were prepared as previously described and rehydrated in PBS or DMEM with 10% FCS (Gibco, Thermofisher Scientific, USA) to a final NIV concentration range of 30 μM in formulation with S-MGB or empty NIV. 25 μL of S-MGB-364, S-MGB-364-NIV, or saline was administered to each nostril (intranasally) of the anaesthetized mice.

### Mycobacterium tuberculosis strain

The hyper-virulent Beijing Mtb strain (HN878) was cultured, titrated, and stored as previously described.^9^

### Generation of murine bone marrow-derived macrophages (BMDMs) and Mtb infection

BMDMs were generated from 8–12 week old C57BL/6 mice as described previously.^10^ BMDMs were cultured overnight for adherence into 96-well plates (Nunc, Denmark) (5 × 10^4^ cells per well). A single-cell suspension of Mtb HN878, from frozen stock, was prepared in DMEM media. 24 h post adherence, BMDMs were infected with Mtb HN878 at MOI 1:5 cfu per well. At 4 h post infection, BMDMs were washed once with culture media to remove extracellular bacteria and lysed. Lysates were plated on 7H10 agar plates containing 10% OADC and 0.5% glycerol for cfu counting to determine bacilli uptake.

### Evaluation of S-MGB-364-induced genotoxicity

24 h post infection, S-MGB-364, S-MGB-364-NIV, and the genotoxicity-inducing agent (10 μM H_2_O_2_) were prepared in DMEM media supplemented with 10% FCS and added to Mtb-infected BMDMs. 4 and 24 h post treatment, BMDMs were harvested and assessed for genotoxicity by flow cytometry using the following antibodies: CD11b (Clone M1/70, PercP-Cy5.5); mouse/human anti-phosphohistone SER-139 H2AX (Clone: H5912-AF488); F4/80 (Clone: BM8-AF647); MHCII (Clone-M5/114.15.2-AF700). Intranuclear expression of γH2AX was detected using BD Pharmingen Transcription Factor Buffer Set (BD Biosciences).

### In vivo Mtb infection and cfu analysis

8–12 week old C57BL/6 mice were anaesthetized and infected intranasally with either 100 cfu/mouse or 1000 cfu/mouse of Mtb HN878 in sterile saline (25 μL per nostril). Mtb-infected mouse lungs were homogenized to measure lung cfu at 5 weeks post infection and to determine bacilli uptake at 1 day post infection as previously described.^9^

### Flow cytometry

Single-cell suspensions were achieved as previously described.^9^ The staining panel was composed of MerTK (Clone: 2B10C42-BV786, BioLegend), CD64 (Clone: X54-5/7-PeCy7, BioLegend), Ly6C (Clone: AL-21-PerCPCy5.5, BD Biosciences), CD11b (Clone: M1/70-V450, BD Biosciences), MHCII (Clone: M5/114.15.2-AF700, BioLegend), CD103 (Clone: M290-PE, BD Biosciences), CD11c (Clone: HL3-APC, BD Biosciences), SiglecF (Clone: E5-2440-APCCy7, BD Biosciences), Ly6G (Clone: 1A8-FITC, BD Biosciences), F4/80 (Clone: BM8-PeCy7, eBiosciences), CD4 (Clone: RM4-5-BV510, BD Biosciences), CD44 (Clone: IM7-PE, BD Biosciences), CD3 (Clone: 500A2-AF700, BD Biosciences), CD62L (Clone: MEL-14-V450, BD Biosciences), CD19 (Clone: 1D3-PerCPCy5.5, BD Biosciences) and CD8 (Clone: 53-6.7-APC, BD Biosciences). Acquisition of samples was conducted using BD LSR Fortessa, and gating strategies are outlined in the Supplementary data (Figure S1 and S2, available at *JAC* Online). Data analysis was performed with FlowJo v10 software (Treestar, Ashland, OR, US).

### Histology and alveolar space assessment

Mtb-infected murine lungs were excised, fixed using 4% phosphate-buffered formalin solution, and stained with haematoxylin and eosin, iNOS, MPO, ARG-1, and anti-CD3, as previously published.^11,12^ Analysis of lung sections and assessment of alveolar space were performed using NIS advanced software on a Nikon (Tokyo, Japan) 90i microscope.

### Cytokine and chemokine determine in lung homogenates

Lung homogenates were centrifuged, and the supernatants were collected. Filtered cell-free lung homogenates were used to detect IL-1α, IGF-1, IL-13, CCL3, CCL5, CXCL1, CXCL2 (R&D Systems), IL-2, TGF-β, IL-4, IL-5, IL-6, IL-12p40, IFN-γ, CCL2 (B&D Biosciences), IL-10, IL-17, IFN-β, TNF and GM-CSF (Biolegend) by enzyme-linked immunosorbent assay (ELISA).

### Statistical analysis of data

All experimental data were analysed using Graph-Pad Prism 8.0.2. Data were calculated as mean ± SEM and the Student’s *t*-test or the one-way ANOVA was used to test for significance.

## Results

### S-MGB-364 interacts strongly with DNA

**Figure 1.**
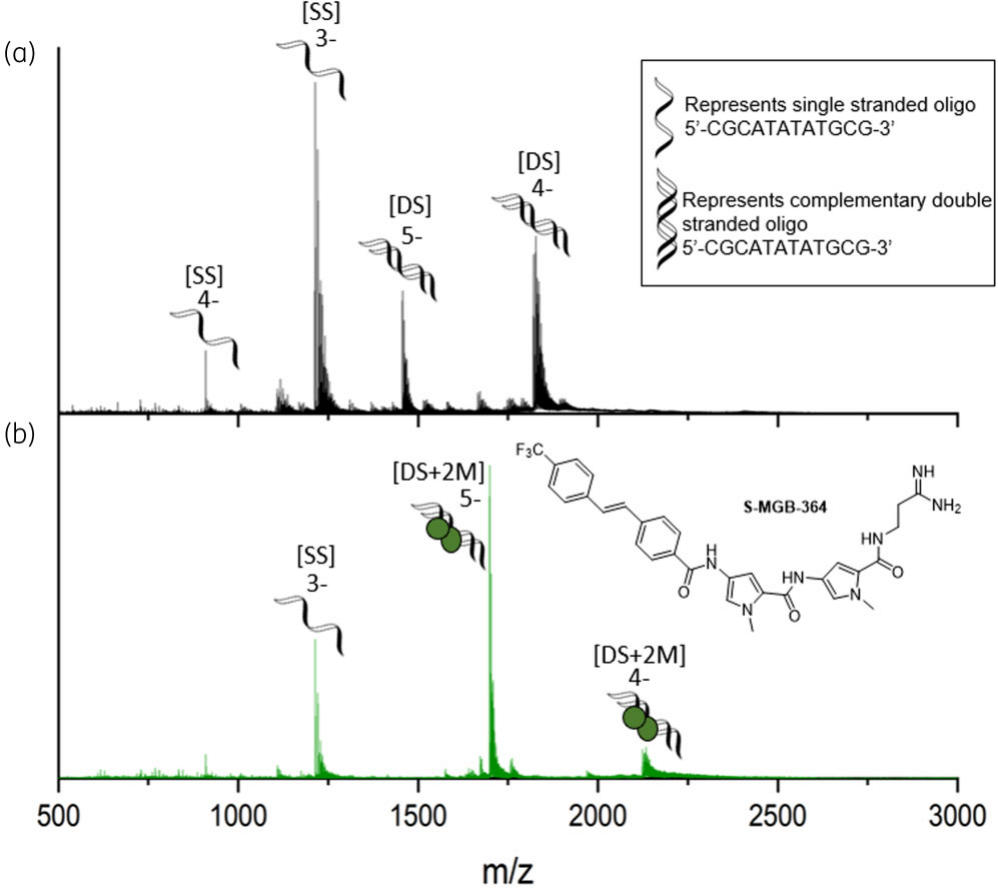
Characterization of S-MGB-364 binding to double-stranded DNA as a dimer by nMS. nMS of DNA sequence 5′-CGCATATATGCG-3′ (9 μM DNA, 100 μM KCl, 1% DMSO) sprayed from ammonium acetate (150 mM, pH 7) in the absence (a) and presence (b) of 100 μM s-MGB. (a) Single-stranded DNA (denoted [SS]) are present in charge states 4− and 3−, and double-stranded DNA (denoted [DS]) are present in charge states 5− and 4−. (b) [SS] is present in charge state 3−. Each [DS] molecule is seen to bind 2×S-MGB molecules (denoted [DS + 2 M]) and is present in charge states 5− and 4−. Figure 1 will appear online in colour and black and white in print.

**Figure 2.**
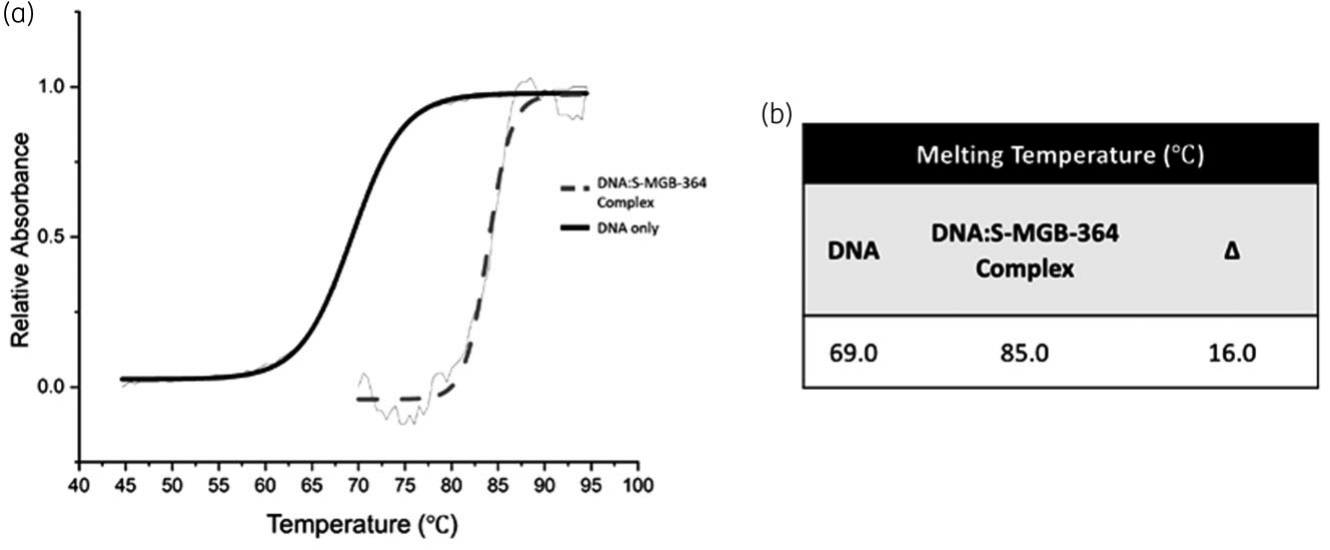
DNA melt curve confirms the ability of S-MGB-364 to bind DNA. (a) Exemplar melt curve from one experimental repeat, visually representing the different melt curves of DNA and the DNA:S-MGB-364 complex. Data has been fitted with a Boltzmann distribution. (b) Melting temperatures of DNA and DNA:S-MGB-364 complex calculated from fitted Boltzmann distributions using OriginPro 2021. All values are an average for *n* = 4 experimental repeats and quoted with an error of ±1°C.

Previously, we observed that S-MGBs bearing an amidine tail group were significantly more active against Mtb than analogues bearing a weakly basic morpholine.^2,7^ Specifically, whilst S-MGB-364 had been identified as a hit compound in our intracellular *in vitro* assay, S-MGB-176, its morpholine tail analogue, was inactive against Mtb.^7^ Consequently, the interaction of both S-MGB-364 and S-MGB-176 with DNA was investigated. Native mass spectrometry (nMS) was used to demonstrate that S-MGB-364 and S-MGB-176 bind to dsDNA, using a short DNA oligo with an AT-rich binding site (5′-CGCATATATGCG-3′). nMS of the DNA in the presence of S-MGB-364 revealed that it is bound to dsDNA exclusively as a dimer [DS + 2M], observed in charge states 5− and 4− (Figure 1, Tables S1 and S2). Similar results were obtained for S-MGB-176 (Figure S3, Tables S3 and S4). Secondly, a DNA thermal melting experiment in the presence and absence of S-MGBs was carried out, using salmon DNA as an example genomic DNA (gDNA) target (Figure 2 and Figure S4). A ΔT_m_ for the DNA:S-MGB-364 complex of 16°C indicated that S-MGB-364 readily binds to and stabilizes DNA. The ΔT_m_ for the DNA:S-MGB-176 complex of only 2.9°C suggests a much weaker interaction with gDNA for S-MGB-176 compared with S-MGB-364 (Table S5). Taken together, the DNA thermal melting and nMS experiments provide conclusive evidence that S-MGB-364 can bind to and stabilize DNA. Moreover, it indicates that the tail group amidine significantly enhances DNA binding.

### S-MGB-364 inhibits Mtb, but not human, topoisomerase I

**Figure 3.**
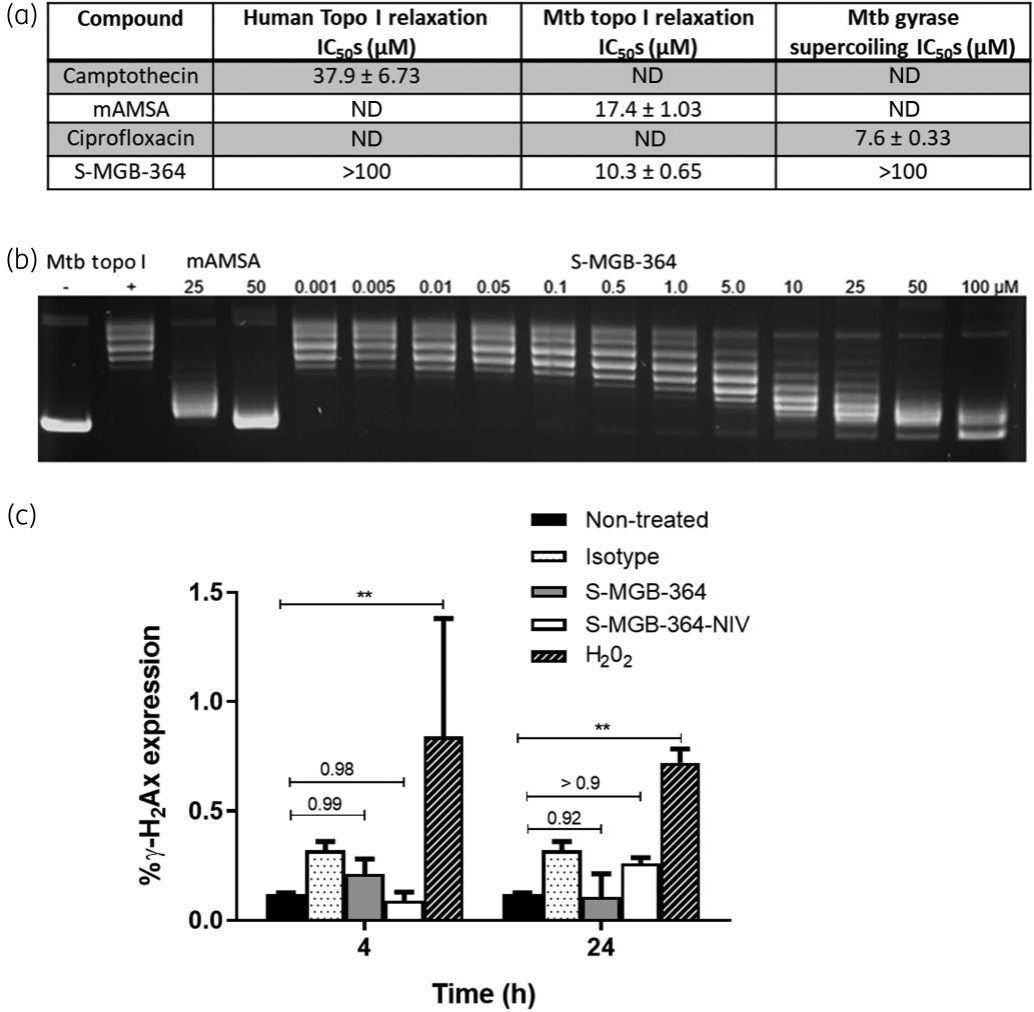
S-MGB-364/S-MGB-364-NIV is non-genotoxic to macrophages and inhibits topoisomerase I. (a) Inhibition of topoisomerases and gyrases by S-MGB-364. Camptothecin, mAMSA, and ciprofloxacin are controls for the human topoisomerase I, Mtb topoisomerase I, and Mtb gyrase enzymes, respectively. These values are presented as mean ± SD with *n* = 2. (b) Example gel illustrating the inhibition of Mtb topoisomerase I relaxation by S-MGB-364. (c) CD11b^+^F4/80^+^MHCII^+^ bone-marrow-derived macrophages were treated with S-MGB-364 (10 μM), S-MGB-364-NIV (10 μM), and the positive control H_2_O_2_ (10 μM) for 4 and 24 h. Intracellular expression of γ-H_2_Ax expression was measured by flow cytometry. (a) Data show mean ± SEM of triplicates. ND, not detected. Two-tailed Student’s *t*-test, **P < 0.01 compared with non-treated.

Whilst S-MGB-364 binding to DNA was confirmed, it was necessary to demonstrate that this could inhibit DNA-centric biological events. To do this, the ability of S-MGB-364 to inhibit the relaxation action of human and Mtb topoisomerase I, and the supercoiling action of Mtb gyrase was evaluated (Figure 3a and b). S-MGB-364 inhibits Mtb topoisomerase I relaxation with an IC_50_ of 10.3 μM, which is more effective than the control compound mAMSA, 17.4 μM. There was no evidence from these experiments that S-MGB-364 inhibits human topoisomerase I relaxation or Mtb gyrase supercoiling. These data confirm the potential of S-MGB-364 to inhibit DNA-centric biological events through binding to DNA.

### S-MGB-364 and S-MGB-364-NIV are non-genotoxic in macrophages

DNA binding drugs could provide safety concerns for mutagenicity and genotoxicity. We used the sensitive molecular marker, γ-H2AX, to assess drug-induced genotoxicity.^13^ We observed no difference in the expression of γ-H2AX in the S-MGB-364 and S-MGB-364-NIV groups relative to the non-treated control at 4 and 24 h post treatment (Figure 3c). Together, these data indicate that S-MGB-364 and S-MGB-364-NIV do not induce genotoxicity in macrophages.

### Intranasal S-MGB-364 and S-MGB-364-NIV administration results in decreased lung burden in Mtb-infected mice

**Figure 4.**
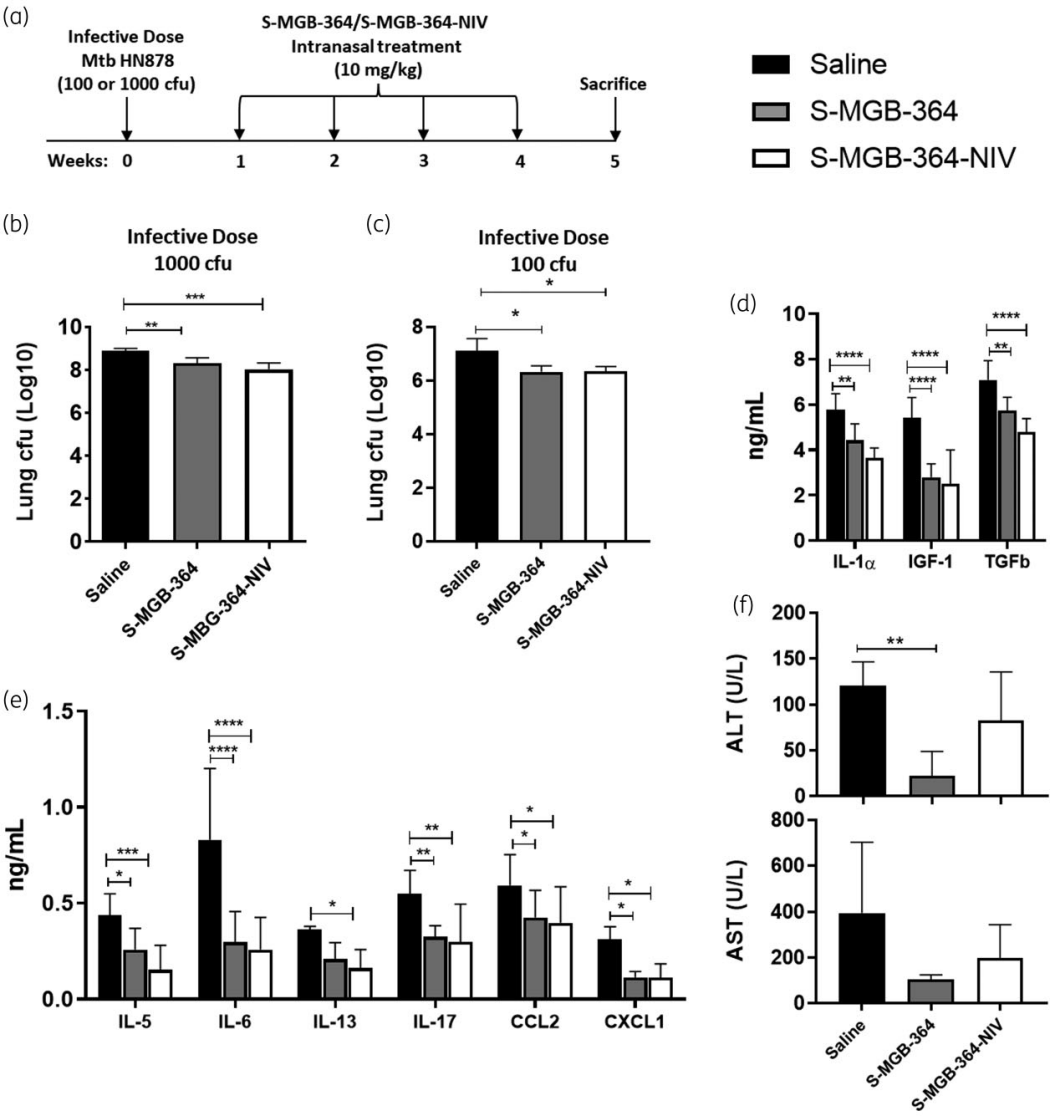
S-MGB-364 and S-MGB-364-NIV treatment is non-toxic and reduced the mycobacterial lung burden in HN878 Mtb-infected mice. (a) C57BL/6 mice (*n* = 6 per group) were infected with Mtb HN878 via intranasal challenge. At 1, 2, 3 and 4 weeks post-infection, mice were intranasally treated with 10 mg/kg of S-MGB-364, S-MGB-364-NIV, or saline. Mice were sacrificed at 5 weeks post-infection and lungs were isolated and homogenized for cfu enumeration. Bacterial load (cfu) measured in lungs of mice infected with an infective dose of (b) 1000 cfu and (c) 100 cfu Mtb HN878 and intranasally treated with S-MGB-364, S-MGB-364-NIV, or saline. (d and e) Supernatants from lung homogenates of mice that were infected with 100 cfu Mtb HN878 were collected for analysis of cytokine/chemokine concentrations by ELISA. (f) Liver aspartate transaminase (AST), and alanine transaminase (ALT) levels were measured in the sera of Mtb-infected (100 cfu) mice treated intranasally with S-MGB-364, S-MGB-364-NIV, or saline. Data are shown as mean ± SEM. Students *t*-test or one-way ANOVA: *P < 0.05, **P < 0.01, and ***P < 0.001.

Previously, we showed that treatment of HN878-infected macrophages with S-MGB-364 reduced the intracellular growth of Mtb.^7^ To extend this finding, S-MGB-364 and encapsulated S-MGB-364-NIV were administered intranasally to Mtb-infected mice at 1, 2, 3, and 4 weeks post infection (Figure 4a). Importantly, and in agreement with our *in vitro* data, treatment comprising of either S-MGB-364 alone or S-MGB-364-NIV was less toxic (Figure 4f) and resulted in a significant reduction (∼1 log) in the lung HN878 burden relative to the control saline group during high dose and a more standard dose of HN878 infection (Figure 4b and c, respectively). Taken together, these data provide *in vivo* proof of concept for S-MGB-364 as a potential anti-TB agent.

### Intranasal treatment with S-MGB-364 and S-MGB-364-NIV reduced proinflammatory cytokine responses in Mtb-infected mice

To assess the activity of S-MGB-364 on lung inflammation, mice were infected with 100 cfu of HN878. After the establishment of Mtb infection, and treatment with S-MGB-364 and S-MGB-364-NIV, cytokine and chemokine levels were measured in lung homogenates by ELISA. S-MGB-364 and S-MGB-364-NIV significantly reduced the levels of IL-1α, IGF-1, TGF-β, IL-5, IL-6, IL-13, IL-17; and the chemokines, CCL2 and CXCL1, relative to the control group (Figure 4d and e). Further, IL-4, IL-12p40, IFN-γ, GM-CSF, TNF, CCL3, CCL5, and CXCL2 were similar to the control group (data not shown). Together, these results indicate a reduction in the lung proinflammatory response following treatment with S-MGB-364 in Mtb-infected mice. Further, this effect is enhanced by the encapsulation of S-MGB-364 within NIVs; however, this difference was non-significant.

### S-MGB-364 alters the recruitment of host lymphoid cells, reduces neutrophils, and recruits interstitial macrophages in the lung during Mtb infection in mice and does not affect mouse lung histopathology

**Figure 5.**
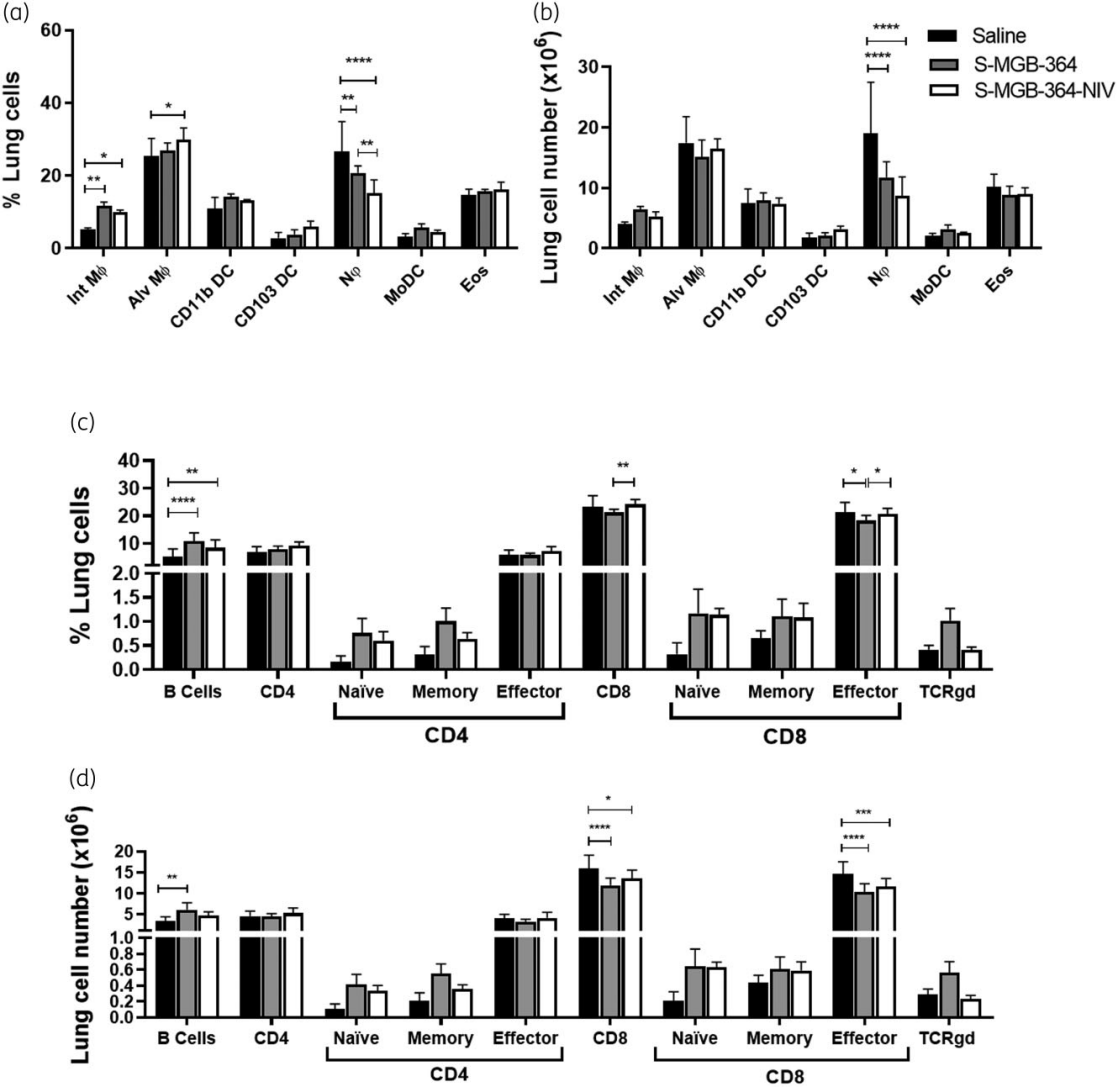
Increased lung macrophage and B cell recruitment and decreased CD8^+^ T cells and neutrophil recruitment in S-MGB-364- and S-MGB-364-NIV-treated mice following Mtb HN878 infection. C57BL/6 mice (*n* = 6 per group) were infected with 100 cfu of Mtb HN878 strain via intranasal challenge. At 1, 2, 3 and 4 weeks post infection, mice were intranasally treated with 10 mg/kg of S-MGB-364, S-MGB-364-NIV, and saline. Mice were sacrificed at 5 weeks post-infection and cellular infiltration was analysed from lung cell suspensions by flow cytometry. (a) Percentage and (b) total cell numbers of myeloid populations, (c) percentage and (d) total cell numbers of lymphoid populations from lung single cell suspensions of mice treated with S-MGB-364, S-MGB-364-NIV or saline. Alveolar macrophages (Alv MΦ) = CD64^+^SiglecF^+^CD11c^+^; interstitial macrophages (Int. MΦ) = MERTK^+^CD64^+^CD11c^−^SiglecF^−^; CD103 dendritic cells (DC) = MHCII^+^CD11c^+^CD103^+^CD11b^−^, CD11b DC = MHCII^+^CD11c^+^CD103^−^CD11b^+^; neutrophils (Nφ) = LY6G^+^CD11b^+^; monocyte-derived dendritic cells (MoDC) = CD64^+^ CD11b^+^CD11c^+^; eosinophils (Eos) = CD64^−^SiglecF^+^CD11b^+^; B cells = CD19^+^CD3^−^; CD8^+^ T cells = CD3^+^CD4^−^CD8^+^; CD4^+^ T cells = CD3^+^CD4^+^CD8^−^; naive T cells = CD62L^+^CD44^−^; memory T cells = CD62L^+^CD44^+^; effector T cells = CD62L^−^CD44^+^; TCRγδ Cells = TCRγδ^+^CD3^+^. Data shown as mean ± SEM. One-way ANOVA: *P < 0.05, **P < 0.01; ***P < 0.001, ****P < 0.0001.

**Figure 6.**
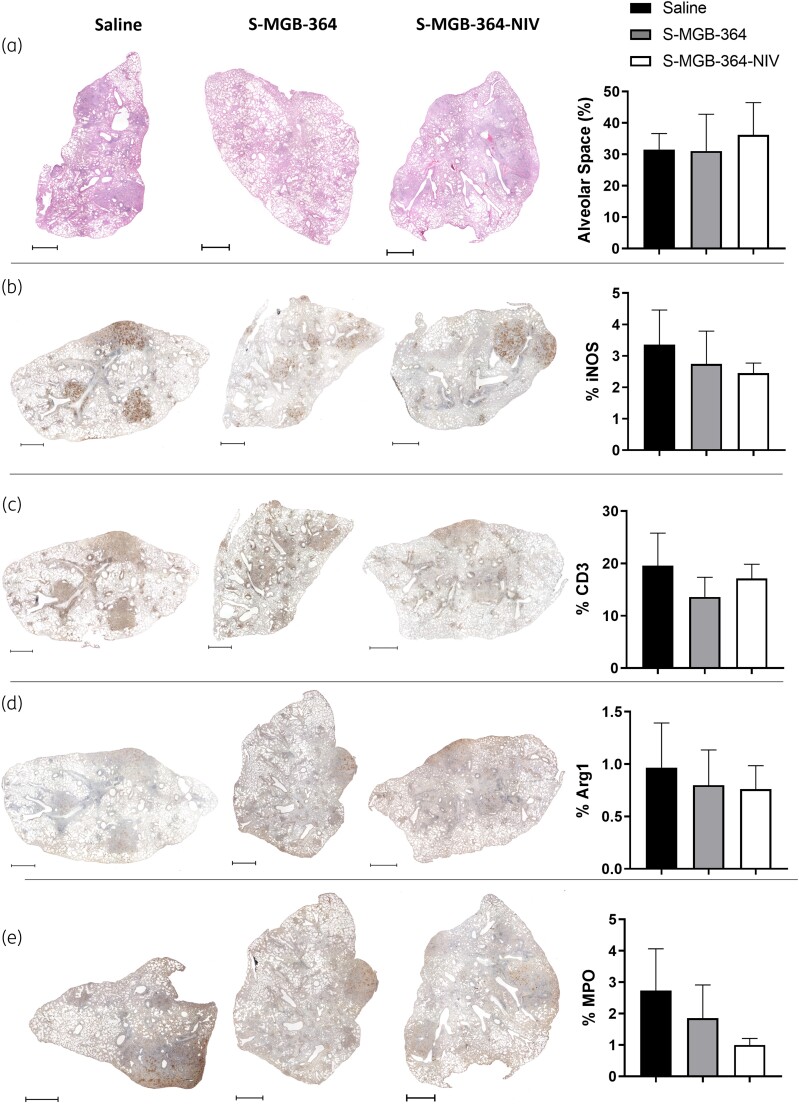
S-MGB-364 and NIV encapsulation did not affect pulmonary histopathology. C57BL/6 mice (*n* = 6 per group) were infected with 100 cfu of Mtb HN878 strain via intranasal challenge. At 1, 2, 3 and 4 weeks post-infection, mice were intranasally treated with 10 mg/kg of S-MGB-364, S-MGB-364-NIV, or saline. Mice were sacrificed at 5 weeks post-infection and lungs were collected in 4% formalin for histopathology analysis. Percentage of (a) alveolar space, (b) iNOS, (c) CD3, (d) Arg1, and (e) MPO. Data show mean ± SEM. Scale bars represent 1000 μm. Magnification = 20×.

We further investigated the effect of S-MGB-364 on immune cell recruitment in the lungs during HN878 infection using flow cytometry. At 5 weeks post infection, S-MGB-364 increased the recruitment of interstitial macrophages whilst decreasing the frequency of neutrophils within the lungs (Figure 5a). Additionally, S-MGB-364 treatment resulted in an increased percentage of B cells as well as a decreased percentage of effector CD8^+^ cells in the lung when compared with the saline-only group (Figure 5c). However, when calculated for total cell numbers, only the reduction in neutrophils and CD8^+^ effector cells were significantly reduced (Figure 5b and d). Histopathological assessment of the lung sections showed that the percentage free alveolar space, iNOS, CD3, and Arg1 levels were similar to the control group (Figure 6a–d). In contrast, the neutrophilic marker myeloperoxidase (MPO) showed a decreased trend in the S-MGB-364-NIV group (*P* = 0.515) (Figure 6e). Together, these data indicate the possible immunomodulation of lung tissue upon S-MGB-364 administration during Mtb HN878 infection.

## Discussion

Previously, we reported the intracellular antimycobacterial activity of two S-MGBs; S-MGB-362 and S-MGB-364.^7^ Further, we showed that NIVs improved compound efficacy whilst being non-toxic in macrophages.^7^ However, the DNA binding, mutagenicity, as well as the *in vivo* immunological, and antimycobacterial properties of these compounds remained unknown. S-MGBs are hypothesized to bind to pathogen DNA in a non-specific, non-covalent manner.^1–3^ This association dysregulates various DNA-centric biochemical events by inhibiting DNA–protein interactions. Previous studies have highlighted these DNA-associated mechanisms in antibacterial,^4^ antiparasitic,^3^ and antifungal models;^2^ however, the mechanism of action had not been validated for anti-TB S-MGBs. Herein we show, for the first time, that S-MGB-364 binds strongly to and stabilizes DNA, using DNA thermal melting and nMS. Further, we demonstrated that S-MGB-364 binds as a dimer to DNA, similar to the natural product, distamycin. Moreover, we showed that the interaction between S-MGB-364 and DNA can lead to inhibition of DNA-centric biochemical events relevant to anti-Mtb activity, such as the action of topoisomerase I. Previous studies have shown that the inhibition of Mtb topo I through the action of small molecules can arrest Mtb growth and can potentially be used as adjunctive anti-TB therapies.^14,15^ Here, we demonstrated that S-MGB-364 inhibited Mtb topoisomerase relaxation, and not human topoisomerase relaxation or Mtb gyrase supercoiling; however, we do not assert this is the only mechanism of action of S-MGB-364. Instead, we speculate that S-MGB-364 will inhibit a range of different DNA–protein interactions, as has been found for the anti-Gram-positive S-MGB, MGB-BP-3.^5^

Mutagenicity is a significant concern for any drug with a DNA-centred mechanism of activity.^16–18^ In our study, we observed a similar low expression of γ-H2AX with S-MGB-364 and the S-MGB-NIV formulation as compared with non-treated macrophages, indicating that S-MGB-364 is non-genotoxic to mammalian cells. These data correlate with our previous studies on cell viability where we showed that macrophages infected with Mtb HN878 remained viable following 5 days of treatment with S-MGB-362 and S-MGB-364, both as a free compound and in the NIV formulation.^7^

This is first reported study of S-MGBs against Mtb *in vivo*. Previous studies have highlighted the ability of intranasally administered anti-TB compounds to directly target lung macrophages whilst being less toxic relative to oral administration. Furthermore, the intranasal administration of vaccines or drugs against TB is reported to be more effective compared with other routes of administration.^19,20^ Our data indicated that oral S-MGB-364 treatment induced a slight but non-significant reduction of cfu in the lungs (1.07-fold) and spleen (1.09-fold) (Figure S5a and b). Additionally, orally administered S-MGB-364 slightly increased liver transaminases (AST and ALT) relative to the saline control (Figure S5c and d). However, this increase was statistically non-significant. Informed by these, as well as our *in vitro* data, we elected to directly target the lungs, the site of Mtb infection through the intranasal administration of S-MGB-364 and its NIV encapsulation. Interestingly, intranasal S-MGB-364 administration resulted in decreased serum liver transaminase levels and had a greater reduction on lung Mtb burden, highlighting its superiority to traditional oral administration (Figure 4b, c and f). However, we noted that NIVs did not have an added antimicrobial benefit that could be attributed to different factors, such as dosing and variable delivery efficiency to Mtb-infected areas.^21^ Despite this, and of note, we demonstrated that 4 weekly treatments with 10 mg/kg S-MGB-364 and S-MGB-364-NIV were less toxic as measured by liver transaminases in comparison with the control saline group (Figure 4f) and reduced the lung burden by ∼1 log in a physiological infective dose and high dose Mtb infection model, respectively. Additionally, the spleen burdens were unaffected following S-MGB-364 treatment (Figure S6). Daily intranasal administration of S-MGB-364 may further improve compound efficacy. However, due to both technical and physiological limitations of such a regimen (i.e. daily anaesthesia) compounded with the risk of adverse animal welfare outcomes (accidental mortality due to repeated intranasal administration),^22,23^ daily administration was not considered. In the future, nebulized forms of S-MGB-364 could be developed to further assess the effect of daily treatment whilst avoiding the risks detailed above. Despite this limitation, these data highlight the anti-TB potential of MGBs.

Whilst S-MGB-364 treatment reduced the lung bacterial burden, it did not ameliorate lung tissue pathology, nor did it have any effect on the l-arginine metabolic pathway as shown by iNOS or arginase-1 expression in lung tissues. Nevertheless, significant changes in lung cytokine levels were observed after treatment with S-MGB-364. The secretion of IL-1α, IGF-1, TGFβ, IL-5, IL-6, IL-13, IL-17; and the chemokines, CCL2 and CXCL1, was significantly reduced. Furthermore, when incorporated into NIVs, the cytokine responses were either the same as with S-MGB-364 treatment alone or lower in the case of IL-1α and TGFβ. IL-1α, IL-6, and IL-17 are essential cytokines for protection against Mtb.^24^ Therefore, a reduction in these cytokines may result in increased susceptibility to Mtb. CCL2 and CXCL1 are known to affect T cell and neutrophil recruitment,^24^ and a reduction in their levels would explain the reduction of these lung cell populations in this study. Neutrophils are associated with excess tissue immunopathology and in some cases can provide a permissive environment for Mtb bacterial growth, from which we can infer that the reduction in neutrophils would be a beneficial effect.^25^

In conclusion, this study demonstrates that S-MGB-364 binds strongly to DNA as a dimer, inhibits Mtb topoisomerase I relaxation, and is non-genotoxic to mammalian cells. However, further mechanistic screening to determine the specific biological mode of action of these compounds will be necessary. Furthermore, S-MGB-364 is active *in vivo* against Mtb, but future studies of its pharmacokinetics, including different delivery systems, are required to produce more-active formulations. Nonetheless, S-MGB-364 holds promise as an anti-TB compound, and we have provided *in vivo* proof of concept in mice of the S-MGB class more generally.

## Supplementary Material

dkac001_Supplementary_Data

## Supplementary data

Tables S1 to S5 and Figures S1 to S6 are available as Supplementary data at *JAC* Online.
