# Supplementary material for: Intranasally administered S-MGB-364 displays antitubercular activity and modulates the host immune response to Mycobacterium tuberculosis infection
Source: J Antimicrob Chemother. 2022 Jan 25;77(4):1061–71. doi: 10.1093/jac/dkac001 (PMC8969509; doi:10.1093/jac/dkac001)
Supplement: dkac001_Supplementary_Data [file dkac001_supplementary_data.docx]

**Supplementary data**

**Intranasally administered S-MGB-364 displays anti-TB activity and modulates the host immune response to *Mycobacterium tuberculosis* infection**

***Supplementary Materials***

Nathan S. KIESWETTER^1,2‡^, Mumin OZTURK^1,2‡^, Lerato HLAKA^1,2‡^, Julius Ebua CHIA^1,2^, Ryan J. O. NICHOL^4^, Jasmine M. CROSS^4^, Leah M. C. MCGEE^4^, Izaak TYSON-HIRST^4^, Rebecca BEVERIDGE^4^, Frank BROMBACHER^1,2,3^, Katharine C. CARTER^5^, Colin J. SUCKLING^4^, Fraser J. SCOTT^4^, and Reto GULER^1,2,3^*

^‡^These authors contributed equally to this work.

^1^International Centre for Genetic Engineering and Biotechnology, Cape Town Component, Cape Town 7925, South Africa.

^2^Department of Pathology, University of Cape Town, Institute of Infectious Diseases and Molecular Medicine (IDM), Division of Immunology and South African Medical Research Council (SAMRC) Immunology of Infectious Diseases, Faculty of Health Sciences, University of Cape Town, Cape Town 7925, South Africa.

^3^Wellcome Centre for Infectious Diseases Research in Africa (CIDRI-Africa), Institute of Infectious Disease and Molecular Medicine (IDM), Faculty of Health Sciences, University of Cape Town, Cape Town 7925, South Africa.

^4^Department of Pure and Applied Chemistry, University of Strathclyde, Glasgow, G1 1XL, Scotland.

^5^Strathclyde Institute of Pharmacy of Biomedical Sciences, University of Strathclyde, Glasgow, G1 1XL, Scotland.

^‡^***Correspondence***: Tel: +27-21-4066033; Fax: +27-86-6407594; ***E-mail***: [reto.guler@uct.ac.za](mailto:reto.guler@uct.ac.za)

***Keywords***: Minor groove binder*, Mycobacterium tuberculosis,* S*-*MGB-364, non-ionic surfactant vesicles, NIV, mice, macrophage, host immune response, HN878.


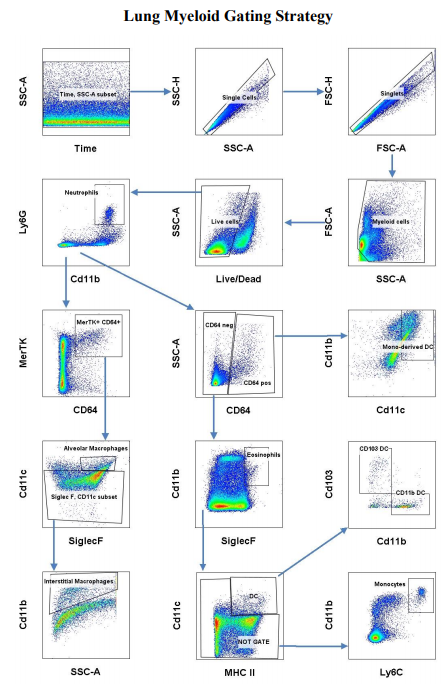


Figure S1: **Myeloid Gating Strategy**


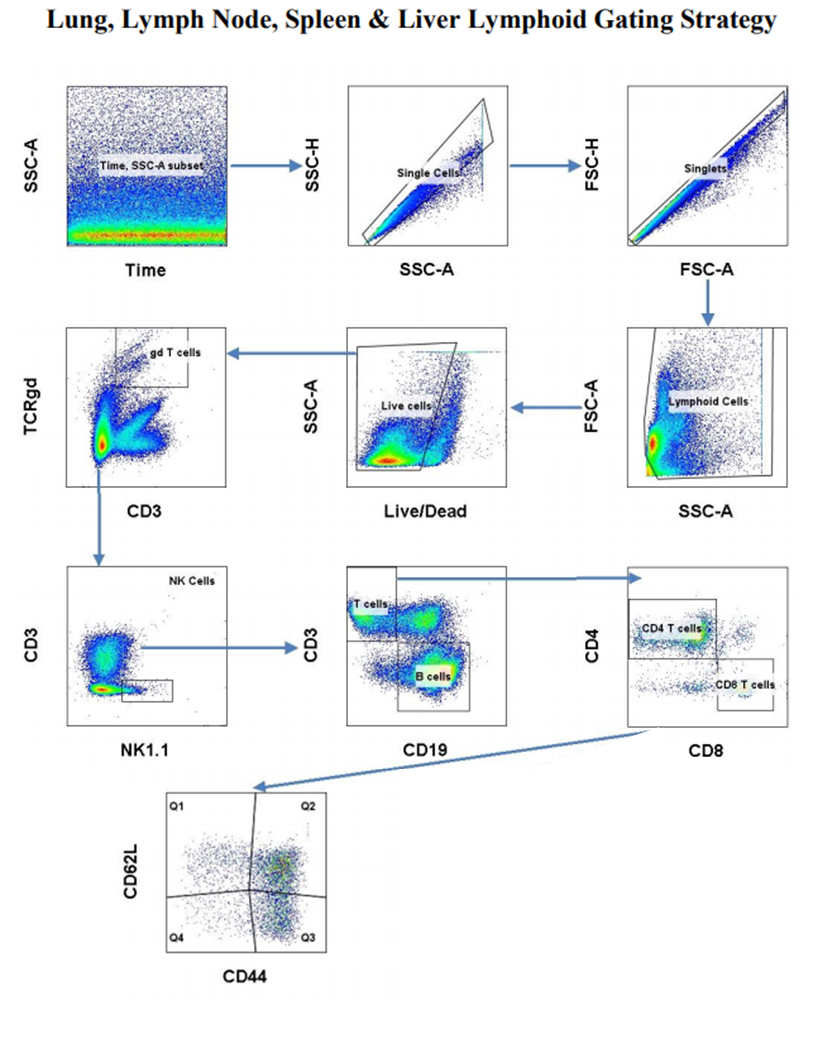


Figure S2: **Lymphoid Gating Strategy**

Table S1: **SI Table with DNA and DNA:S-MGB-364 complex graph model details**. y = A2 + (A1-A2)/(1 + exp((x-x0)/dx))

| MGB  364 | Model | A1 | A2 | X_0_ | dx | Reduced Chi-Sqr | R-Square (COD) | Adj. R-Square |
| --- | --- | --- | --- | --- | --- | --- | --- | --- |
| DNA 1A | Boltzmann | 0.03975 ± 0.00253 | 1.01102 ± 0.00237 | 68.9906 ± 0.049 | 2.86126 ± 0.0431 | 1.74016E-4 | 0.99908 | 0.99905 |
| DNA 1B | Boltzmann | 0.14664 ± 0.00495 | 0.9964 ± 0.00441 | 68.52479 ± 0.11475 | 3.50794 ± 0.10284 | 5.25584E-4 | 0.99613 | 0.99601 |
| COM 1A | Boltzmann | -0.16415 ± 0.01472 | 1.12428 ± 0.01993 | 85.16257 ± 0.11123 | 1.01212 ± 0.09666 | 0.00504 | 0.98617 | 0.98528 |
| COM 1B | Boltzmann | -0.04082 ± 0.00825 | 1.05579 ± 0.01134 | 85.03737 ± 0.08062 | 1.23724 ± 0.07032 | 0.00145 | 0.99424 | 0.99387 |
| DNA 2A | Boltzmann | 0.02607 ± 0.00189 | 0.97877 ± 0.00184 | 69.31254 ± 0.0373 | 2.76381 ± 0.03277 | 1.01805E-4 | 0.99945 | 0.99943 |
| DNA 2B | Boltzmann | 0.13309 ± 0.00412 | 0.98338 ± 0.00392 | 69.06217 ± 0.09416 | 3.11378 ± 0.08344 | 4.29794E-4 | 0.99697 | 0.99687 |
| COM 2A | Boltzmann | -0.04115 ± 0.01423 | 0.97488 ± 0.01759 | 83.91085 ± 0.1421 | 1.24996 ± 0.12418 | 0.00384 | 0.98281 | 0.98169 |
| COM 2B | Boltzmann | -0.00883 ± 0.00972 | 1.07186 ± 0.01244 | 84.3825 ± 0.08415 | 1.00131 ± 0.07316 | 0.00206 | 0.99205 | 0.99154 |

Table S2: **Calculated and measured masses for each species observed in Figure 1 for DNA sequence 5’-CGCATATATGCG-3’and S-MGB-364.** For all species, the expected mass corresponds to the left-hand side of the m/z peaks.

| Species | m/z value | Calculated mass of neutral species (Da) |
| --- | --- | --- |
| Single Stranded [SS] | 3- : 1214.1  4- : 910.3 | (1214.1*3) + 3 = 3645.3  (910.3*4) + 4 = 3645.2 |
| Double Stranded [DS] | 4- : 1821.7  5- : 1457.2 | (1821.7*4) + 4 = 7290.8  (1457.2*5) + 5 = 7291 |
| *Double Stranded + 2 x S-MGB-364 [DS+2M]* | 4- : 2124.2  5- : 1699.2 | (2124.2*4) + 4 = 8500.8  (1699.2*5) + 5 = 8501.0 |


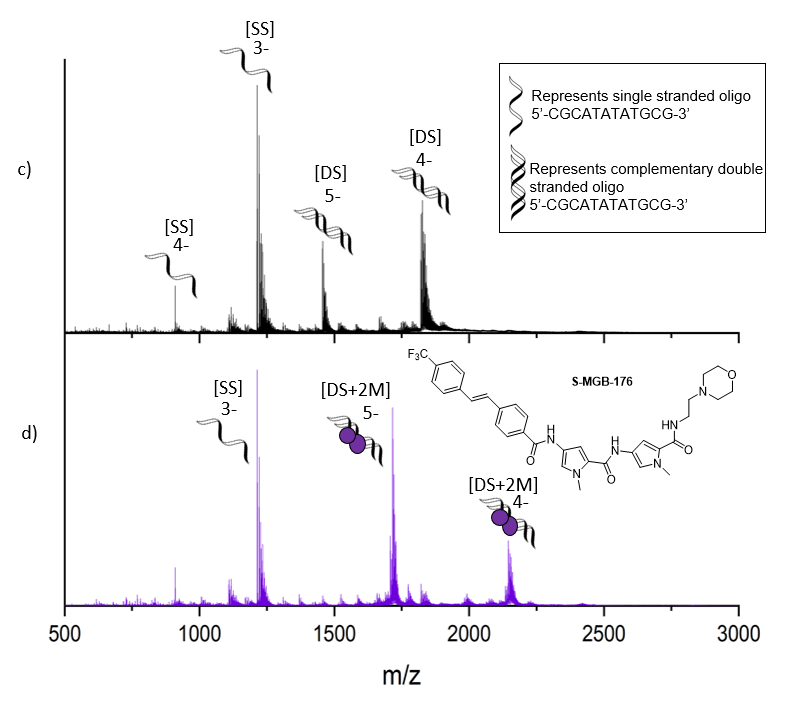


Figure S3: **Characterization of S-MGB-176 binding to double-stranded DNA as a dimer by nMS.** nESI-MS of DNA sequence 5’-CGCATATATGCG-3’ (9 μM DNA, 100 μM KCl, 1% DMSO) sprayed from ammonium acetate (150 mM, pH 7) in the absence **A**) and presence **B**) of 100 μM s-MGB. **A)** Single-stranded DNA (denoted [SS] are present in charge states 4- and 3-, and double-stranded DNA denoted [DS]) are present in charge states 5-- and 4-. **B)** [SS] is present in charge state 3-. Each [DS] molecule is seen to bind 2xS-MGB molecules (denoted [DS+2M]) and is present in charge states 5- and 4-.

Table S3: **SI Table with DNA and DNA:S-MGB-176 complex graph model details.** y = A2 + (A1-A2)/(1 + exp((x-x0)/dx))

|  | Model | A1 | A2 | X_0_ | dx | Reduced Chi-Sqr | R-Square (COD) | Adj. R-Square |
| --- | --- | --- | --- | --- | --- | --- | --- | --- |
| DNA 1A | Boltzmann | 0.00188 ± 0.00146 | 0.99267 ± 0.00139 | 69.23323 ± 0.02818 | 2.92078 ± 0.02482 | 5.80404E-5 | 0.9997 | 0.9997 |
| DNA 1B | Boltzmann | 0.03414 ± 0.00346 | 0.98623 ± 0.00275 | 68.90337 ± 0.06193 | 3.11959 ± 0.05512 | 2.19107E-4 | 0.99872 | 0.99867 |
| COM 1A | Boltzmann | -0.02373 ± 0.0064 | 1.05175 ± 0.00636 | 71.64699 ± 0.09818 | 2.13421 ± 0.08559 | 0.00127 | 0.9949 | 0.99473 |
| COM 1B | Boltzmann | 0.00455 ± 0.00501 | 0.98588 ± 0.00503 | 71.78587 ± 0.09112 | 2.55434 ± 0.07998 | 7.03314E-4 | 0.99644 | 0.99632 |
| DNA 2A | Boltzmann | 0.01452 ± 0.00222 | 0.97424 ± 0.00212 | 69.2806 ± 0.04452 | 2.96255 ± 0.03926 | 1.33033E-4 | 0.99927 | 0.99925 |
| DNA 2B | Boltzmann | 0.12367 ± 0.00398 | 0.99618 ± 0.0036 | 68.58859 ± 0.08741 | 3.14915 ± 0.07745 | 3.84149E-4 | 0.99741 | 0.99733 |
| COM 2A | Boltzmann | 0.12027 ± 0.00483 | 0.99981 ± 0.00537 | 71.87869 ± 0.09691 | 2.00451 ± 0.08429 | 9.17892E-4 | 0.99458 | 0.99441 |
| COM 2B | Boltzmann | -0.02473 ± 0.00506 | 0.97571 ± 0.00586 | 72.19765 ± 0.10564 | 2.85756 ± 0.0929 | 8.54398E-4 | 0.99574 | 0.99561 |

Table S4*:* **Calculated and measured masses for each species observed in Figure S3 for DNA sequence 5’-CGCATATATGCG-3’ and S-MGB-176.** For all species, the expected mass corresponds to the left-hand side of the m/z peaks.

| Species | m/z value | Calculated mass of neutral species (Da) |
| --- | --- | --- |
| Single Stranded [SS] | 3- : 1214.1  4- : 910.3 | (1214.1*3) + 3 = 3645.3  (910.3*4) + 4 = 3645.2 |
| Double Stranded [DS] | 4- : 1821.7  5- : 1457.2 | (1821.7*4) + 4 = 7290.8  (1457.2*5) + 5 = 7291.0 |
| *Double Stranded + 2 x S-MGB-176 [DS+2M]* | 4- : 2146.0  5- : 1716.6 | (2146.0*4) + 4 = 8588.0  (1716.6*5) + 5 = 8588.0 |


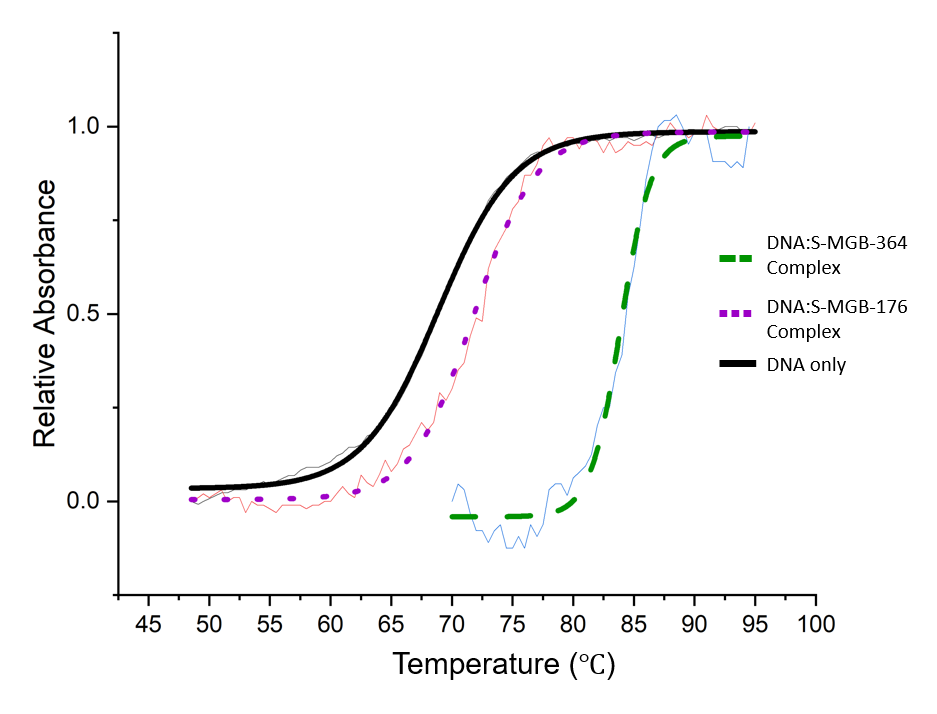


Figure S4: **DNA melt curve comparing the ability of S-MGB-364 and S-MGB-176 to bind DNA**

Table S5: **Melting temperatures of DNA and DNA:S-MGB complexes**. All values quoted are within an error of ±1℃

| Melting Temperature (℃) | | | |
| --- | --- | --- | --- |
| MGB | DNA | DNA:S-MGB Complex | Δ |
| S-MGB-176 | 69.0 | 71.9 | 2.9 |
| S-MGB-364 | 69.0 | 85.0 | 16.0 |


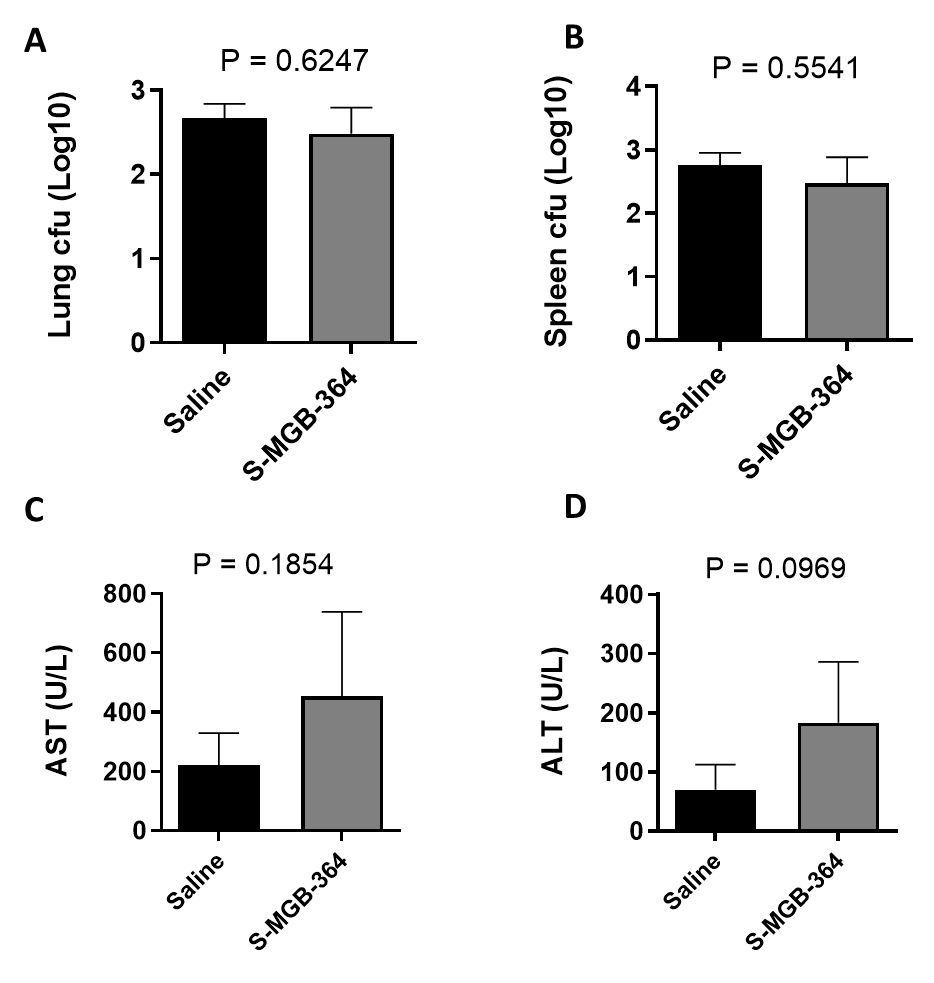


Figure S5: **Oral S-MGB-364 did not affect cfu burden and was non-toxic assessed by liver transaminases.** C57BL/6 mice (n = 6 per group) were infected with 100 cfu of Mtb HN878 via oral challenge. At 1-, 2-, 3- and 4-weeks post-infection, mice were orally treated with 10 mg/kg of S-MGB-364 or saline. Mice were sacrificed at 5-weeks post-infection and **A**) lungs and **B**) spleens were isolated and homogenized for cfu enumeration. Liver transaminases, **C**) aspartate transaminase (AST), and **D**) alanine transaminase (ALT) were assessed in sera of Mtb-infected mice at 5-weeks post-infection.

Figure S6: **Spleen burden was unaffected by intranasal S-MGB-364 treatment.** C57BL/6 mice (n = 6 per group) were infected with 1000 cfu of Mtb HN878 via intranasal challenge. At 1-, 2-, 3- and 4-weeks post-infection, mice were intranasally treated with 10 mg/kg of S-MGB-364, S-MGB-364-NIV, or saline. Mice were sacrificed at 5-weeks post-infection and the spleens were isolated and homogenized for cfu enumeration.
